# Monoclonal antibodies targeting CD38 in hematological malignancies and beyond

**DOI:** 10.1111/imr.12389

**Published:** 2016-02-10

**Authors:** Niels W. C. J. van de Donk, Maarten L. Janmaat, Tuna Mutis, Jeroen J. Lammerts van Bueren, Tahamtan Ahmadi, A. Kate Sasser, Henk M. Lokhorst, Paul W. H. I. Parren

**Affiliations:** ^1^ Department of HematologyVU University Medical CenterAmsterdamthe Netherlands; ^2^ GenmabUtrechtthe Netherlands; ^3^ Janssen Research and DevelopmentSpring HousePAUSA; ^4^ Department of Cancer and inflammation ResearchInstitute of Molecular MedicineUniversity of Southern DenmarkOdenseDenmark; ^5^ Department of Immunohematology and Blood TransfusionLeiden University Medical CenterLeidenthe Netherlands

**Keywords:** CD38, therapeutic antibody, daratumumab, isatuximab, multiple myeloma, cancer

## Abstract

CD38 is a multifunctional cell surface protein that has receptor as well as enzyme functions. The protein is generally expressed at low levels on various hematological and solid tissues, while plasma cells express particularly high levels of CD38. The protein is also expressed in a subset of hematological tumors, and shows especially broad and high expression levels in plasma cell tumors such as multiple myeloma (MM). Together, this triggered the development of various therapeutic CD38 antibodies, including daratumumab, isatuximab, and MOR202. Daratumumab binds a unique CD38 epitope and showed strong anti‐tumor activity in preclinical models. The antibody engages diverse mechanisms of action, including complement‐dependent cytotoxicity, antibody‐dependent cellular cytotoxicity, antibody‐dependent cellular phagocytosis, programmed cell death, modulation of enzymatic activity, and immunomodulatory activity. CD38‐targeting antibodies have a favorable toxicity profile in patients, and early clinical data show a marked activity in MM, while studies in other hematological malignancies are ongoing. Daratumumab has single agent activity and a limited toxicity profile, allowing favorable combination therapies with existing as well as emerging therapies, which are currently evaluated in the clinic. Finally, CD38 antibodies may have a role in the treatment of diseases beyond hematological malignancies, including solid tumors and antibody‐mediated autoimmune diseases.

This article is part of a series of reviews covering Immunoglobulins: from genes to therapies appearing in Volume 270 of *Immunological Reviews*.

## CD38: the target

CD38, in the past referred to as T10, was identified in 1980 in a project investigating cell surface molecules of human leukocytes using thymocyte‐specific murine monoclonal antibodies 1. In the early days, CD38 was recognized as a differentiation and activation marker of lymphocytes, later it was used as a phenotypic marker in the classification of T‐ and B‐cell malignancies, and identified as a promising target for antibody‐based therapies. It was also found that CD38 has multiple receptor and enzymatic functions. Here, we bring all these aspects of CD38 together, and give our perspective on CD38‐targeted therapy.

### The CD38 gene

The human CD38 gene is located on chromosome 4 (4p15) and stretches over 62 kb. The gene comprises seven introns, which represent the vast majority (>98%) of the gene, and eight exons that encode the CD38 protein 2. Gene transcription is regulated at multiple levels. The promoter region is characterized by the presence of CpG islands that are controlled by methylation, but lacks canonical TATA and CAAT boxes 3. Potential binding sites for various immunological transcription factors, including those for T cell‐specific transcription factor alpha (TCF‐1α), nuclear factor‐IL‐6 (NF‐IL‐6), and interferon responsive factor‐1 (IRF‐1) are located upstream of these CpG islands 3. Tumor necrosis factor‐alpha (TNFα), for example, has been shown to mediate interferon‐dependent induction of CD38 expression in airway smooth muscle cells 4, and IFNγ upregulates expression and activity of CD38 in human monocytes 5. Another level of transcriptional control is positioned within intron 1, which contains responsive elements for retinoic acid (RA) 6 and peroxisome proliferator‐activated receptor γ (PPARγ) 7. Interestingly, *in vivo* upregulation of CD38 via the RA receptor signaling pathway was observed in patients with promyelocytic leukemia following a single oral administration of RA 6, 8, 9. Finally, the transcription factor E2A mediates CD38 gene transcription in response to environmental signals, such as interleukin‐2 and TLR‐9 ligands 10. Transcription initiation by E2A is affected by the presence of a single nucleotide polymorphism (SNP) at the 5′‐end of intron 1, which is located within a putative E‐box 10, 11, 12. The presence of a guanine (G) instead of a cytosine (C) at this position, which is present in approximately 14% of the healthy Caucasian population, enhances CD38 transcription and results in increased protein expression levels 10, 11. Of note, the frequency of this relatively rare G allele was significantly higher in a subset of chronic lymphocytic leukemia (CLL) patients with markers of poor prognosis 10.

### CD38 protein structure

The human CD38 antigen is a 46‐kDa type II transmembrane glycoprotein. The primary and secondary structure of CD38 exhibits a striking similarity (approximately 35% amino acid identity) to a soluble cyclase that was identified from the mollusk *Aplysia californica* 13, 14. CD38 contains a relatively long C‐terminal extracellular domain (258 aa), a transmembrane region (21 aa) and a short N‐terminal cytoplasmic tail (21 aa) 15. The protein is stabilized by six disulfide bonds and has four potential N‐linked glycosylation sites. The crystal structure of the CD38 extracellular domain has been resolved to 1.9 Å, revealing that the protein's overall topology is similar to the related proteins CD157 and the *Aplysia* ADP‐ribosyl cyclase, except for large structural differences at the two termini 16. Further biochemical observations indicated the existence of a CD38 tetramer on the cell surface 17, 18, 19. Tetramerization was suggested to be required for the CD38 catalytic activity and the localization of CD38 in lipid rafts 20. In addition to the cell membrane‐bound form, a 39‐kDa soluble variant of CD38 has been found in biological fluids 21, and a 78‐kDa soluble form was identified from cells of X‐linked agammaglobulinemia patients 19.

### CD38: the receptor

Early functional studies showed that CD38 regulates weak adhesion events that take place between circulating lymphocytes and endothelial cells 22. This finding was key in identifying a receptor function for CD38. A murine mAb raised against HUVEC blocked CD38‐mediated adhesion of several cell lines to HUVEC 23. By resolving the target of this blocking mAb, CD31 was identified as a ligand for CD38 24. CD31 (also known as PECAM‐1) is a member of the Ig gene superfamily characterized by six Ig‐like domains 25. In addition to its expression on endothelial cells, CD31 is expressed on lymphoid cells (follicle mantle B cells and plasma cells), in the lungs (alveolar ducts, alveoli, and lymphatic vessels), and in the kidney (glomerular cells) 26. The CD38–CD31 interaction does not only play a role in the binding and migration of leukocytes through the endothelial cell wall but also triggers activation and proliferation of human leukocytes 24, 27. Moreover, the adhesion function of CD38 is involved in the differentiation of B cells, which requires heterotypic interactions as a crucial developmental step.

### CD38: the ecto‐enzyme

Next to its receptor function, CD38 has bifunctional ecto‐enzymatic activity 28, 29. The protein has cyclase as well as hydrolase activity. Similar to its *Aplysia* homolog, CD38 uses NAD^+^ as substrate for the formation of cyclic ADP‐ribose (cADPR) and ADPR. Indeed, studies in CD38 knockout mice showed that CD38 is indispensable for NAD^+^‐glycohydrolase activity in the liver and brain 30. In acidic conditions, CD38 in addition catalyzes the generation of nicotinic acid‐adenine dinucleotide phosphate (NAADP^+^) from nicotinamide adenine dinucleotide phosphate (NADP^+^) 29, 31, 32. cADPR, ADPR, and NAADP^+^ are potent second messengers that regulate Ca^2+^ mobilization from the cytosol 33, activating signaling pathways that control various biological processes, such as lymphocyte proliferation 34 and insulin secretion by β‐cells in the pancreas 35, 36, 37. Interestingly, recent studies suggest a pivotal role of CD38‐dependent adenosine production in immune suppression mediated by natural killer (NK) cells 38 and an involvement of CD38 in immune modulation mediated by myeloid‐derived suppressor cells 39.

### CD38 tissue distribution

CD38 is expressed on lymphoid and myeloid cells as well as in a several non‐hematopoetic tissues 40.

Expression of CD38 in hematopoietic cells depends on the differentiation and activation status of the cell. Lineage‐committed hematopoietic cells express the protein, while it is lost by mature cells and expressed again on activated lymphocytes. For example, the majority of medullary thymocytes show expression of CD38, circulating T cells do not express CD38, while expression is induced in activated T cells. CD38 is also expressed on B cells, whereby plasma cells express particularly high levels of CD38. Approximately 80% of resting NK cells and monocytes express CD38 at lower levels, as do various other hematological cell types, including lymph node germinal center lymphoblasts, intrafollicular cells 15, dendritic cells, erythrocytes 41, 42, and platelets 15, 41, 43, 44, 45. Although CD38 is widely expressed on cells of hematopoietic origin, including committed stem cells, the hematopoietic compartment remained unaltered in CD38 knockout mice 30, suggesting that CD38 is not critical to hematopoiesis or lymphopoiesis in mice.

With regard to non‐hematopoietic tissues, CD38 is expressed at low levels in some specific cell types, such as Purkinje cells and neurofibrillary tangles in the brain, epithelial cells in the prostate, β‐cells in the pancreas, osteoclasts in the bone, retinal cells in the eye, and sarcolemma of smooth and striated muscle 40.

## Expression of CD38 in disease

Expression of CD38 has been associated with a number of diseases, including HIV infection 46, 47, autoimmune diseases [e.g. systemic lupus erythematosus 48], type II diabetes mellitus 35, osteoporosis 49, and cancer. Most notably, CD38 is expressed in a large number of hematological malignancies. Expression has been observed particularly in the malignant cells of multiple myeloma (MM) 50 and CLL 51, and was also reported in Waldenström's macroglobulinemia 52, primary systemic amyloidosis 53, mantle cell lymphoma 54, acute lymphoblastic leukemia 55, acute myeloid leukemia 55, 56, NK cell leukemia 57, NK/T‐cell lymphoma 58, and plasma cell leukemia 59.

### Multiple myeloma

MM is a neoplasm of the B‐cell lineage that is characterized by proliferation of plasma cells in the bone marrow. This typically results in anemia and increased bone resorption by osteoclasts, frequently leading to bone fractures or hypercalcemia. MM is further characterized by the production of excess immunoglobulin, usually of an IgG class, (M‐protein) or fragments thereof (kappa or lambda free light chains). Large amounts of free light chains may result in damage to tubular cells in the kidney, leading to acute renal failure.

Over the last decade, the survival of MM patients has significantly improved due to the application of autologous stem cell transplantation in younger patients and the introduction of novel drugs such as the immunomodulatory agents (IMiDs) lenalidomide and pomalidomide, and the proteasome inhibitors (PI) bortezomib and carfilzomib. However, the far majority of patients still relapse even when intensive therapy is combined with IMiDs and PIs 60. In addition, patients who present with high‐risk disease as determined by cytogenetic analysis (especially deletion of chromosome 17p), gene‐expression profiling, or clinical characteristics such as presence of extramedullary disease or circulating tumor cells (plasma cell leukemia) benefit less for the newly available treatments 61. Altogether, this clearly demonstrates that there is a need for additional active agents with distinct mechanisms of action, especially for the relapsed and refractory patients.

Therapeutic antibodies are successfully used in many hematological cancers, but no appropriate antibodies are yet available for MM. However, with various candidates in late‐stage clinical development, monoclonal antibodies are an emerging class of agents for the treatment of MM 62. Therapeutic antibodies generally show a favorable tolerability profile with the primary toxicity targeted to the tumor cell. Depending on the antigen bound, mAbs engage diverse mechanisms of action that differ from existing therapies of MM, including IMiDs and proteasome inhibitors.

CD38 is a highly interesting target for antibody therapy in MM and related disorders, as virtually all MM cells express high levels of CD38 on their cell surface, similar to normal plasma cells 50.

## CD38 antibodies

High expression levels in combination with its role in cell signaling points to CD38 as an attractive therapeutic antibody target, in particular for MM and CD38‐positive NHL. Indeed, various CD38 antibodies have been developed and show strong preclinical and clinical potential.

Most advanced in development is the human anti‐CD38 IgG1 daratumumab, which is in late‐stage clinical studies for the treatment of MM in combination with a number of drugs representing the standard of care. Daratumumab was originally developed by Genmab 63 and licensed by Janssen Biotech in 2012. In 2013, the Food and Drug Administration (FDA) granted Breakthrough Therapy Designation to daratumumab for the treatment of patients with MM who have received at least three prior lines of therapy including a PI and an IMiD, or who are double refractory to a PI and IMiD. The FDA approved daratumumab (Darzalex) for this indication on November 16, 2015 under a priority review. The European Medicines Agency (EMA) also granted a priority review for the daratumumab Marketing Authorization Application. Daratumumab received Orphan Drug Designation from the US FDA and the EMA for the treatment of MM.

In addition to Janssen Biotech, two other companies, Morphosys (Munich, Germany) and Sanofi‐Aventis (Paris, France), have CD38‐specific antibodies in clinical development. Sanofi Oncology has developed isatuximab (SAR650984) (64), a humanized anti‐CD38 antibody [which obtained a chimeric designation under the new 2014 definitions for INN]. Isatuximab is currently being tested in early clinical trials in CD38‐positive hematological malignancies, including MM 64, 65. Morphosys is developing the human CD38 antibody MOR202 that is being investigated as monotherapy and in combination with lenalidomide/dexamethasone or pomalidomide/dexamethasone in a phase I/II study in refractory or relapsed MM.

Furthermore, several CD38‐targeting antibodies have been described that are in preclinical development: Takeda is developing the human anti‐CD38 antibodies Ab79 and Ab19 66, Xencor has a CD3/CD38 bispecific antibody program 67, and Molecular Templates is developing an anti‐CD38 antibody–drug conjugate (MT‐4019).

## Daratumumab, a human monoclonal antibody with broad‐spectrum anti‐tumor activity

### Daratumumab binds to a unique epitope

Daratumumab (also referred to as HuMax‐CD38 and IgG1‐005) is an immunoglobulin G1 kappa (IgG1κ) human monoclonal antibody (mAb) that specifically binds a unique epitope present on the CD38 molecule. Specific and strong binding of daratumumab to CD38 expressed on the cell surface on Daudi cells as well as fresh MM cells was shown 63. BIAcore analysis confirmed that daratumumab binds with high affinity to human CD38, with an affinity constant (*K*_D_) of 4.36 nM (Genmab, data on file). Daratumumab was selected from a panel of 42 CD38‐specific mAbs that were generated with the validated HuMAb technology using human Ab transgenic mice 68. Daratumumab stood out in its exceptional ability to mediate complement‐dependent cytotoxicity (CDC) of CD38‐overexpressing cell lines as well as patient‐derived MM cells 63. Prophylactic treatment with daratumumab induced a remarkably high anti‐tumor efficacy in a Daudi tumor model in mice 63. Daratumumab also induced marked anti‐tumor activity in a MM xenograft model in a late treatment setting, in which daratumumab was given 3 weeks after tumor cell inoculation 63.

Using a constrained peptide approach, the epitope of daratumumab was mapped to two β‐strands containing amino acids 233–246 and 267–280 of CD38. Following the observation that daratumumab does not bind to cynomolgus CD38, CD38 variants incorporating amino acid differences between human and cynomolgus CD38 were generated. Using these variants, specific amino acid residues were identified which are important for daratumumab binding, in particular serine at position 274 63. We speculate that the daratumumab epitope on CD38, as well as the antibody's binding orientation, offer a favorable structural arrangement for hexamer formation, allowing efficient docking of C1q and initiation of the complement pathway (69).

**Figure 1:**
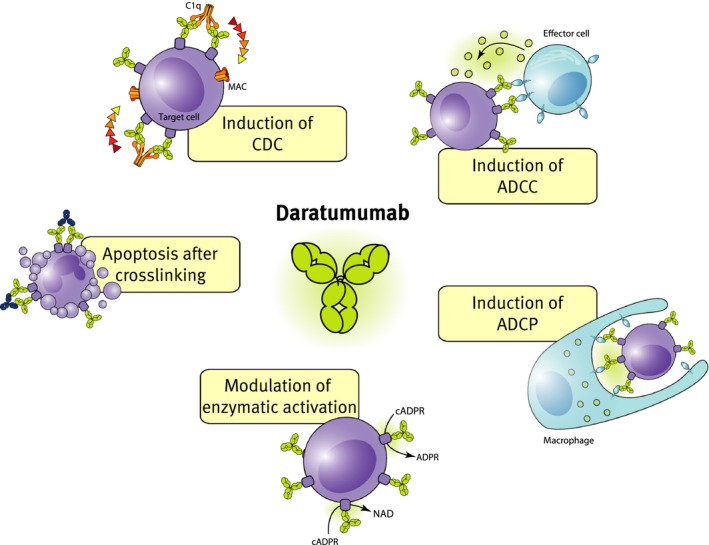
**Mechanisms of action of daratumumab.**

Daratumumab has a broad‐spectrum killing activity. In addition to CDC, the binding of daratumumab to CD38 on the surface of tumor cells has been shown to induce antibody‐dependent cellular cytotoxicity (ADCC), antibody‐dependent cellular phagocytosis (ADCP), tumor cell apoptosis, and modulation of the enzymatic activity CD38 (*Fig.* 1).

### Complement‐dependent cytotoxicity

Complement activation is an extremely potent mechanism of action that is highly epitope dependent and therefore only deployed by a small fraction of antibodies (70 and references therein). Complement activation induces the generation of opsonins, chemoattractants, anaphylatoxins, and membrane attack complexes 71, 72. As mentioned, daratumumab was selected from a panel of CD38 antibodies based on its superior CDC activity *in vitro*. The ability of daratumumab to induce CDC was further demonstrated *in vitro* in various tumor cells and cell lines in the presence of complement‐containing human serum. Daratumumab was also found to induce streamers, which is indicative for induction of CDC 73. Significantly, daratumumab induced killing of freshly isolated MM cells obtained from the bone marrow of previously untreated or refractory MM patients 63. In CDC assays with bone marrow samples from MM patients, a marked heterogeneity in susceptibility of the MM cells to daratumumab was observed, which could not be explained by the extent of previous therapy 74. These differences may be explained by variation in expression levels of CD38 74.

### Antibody‐dependent cell‐mediated cytotoxicity

Observations that clinical response after therapeutic antibody treatment correlated with FcγRIIIa polymorphisms 75, 76 indicated that Fc‐receptor‐dependent mechanisms substantially contribute to the cytotoxic action of therapeutic antibodies against tumors.

In preclinical studies, we have shown that daratumumab triggers Fc‐receptor‐mediated tumor cell killing, including ADCC 63. In *in vitro* assays using PBMCs from healthy donors as effector cells, it was shown that daratumumab mediated dose‐dependent ADCC against Daudi cells and a panel of drug‐sensitive and drug‐resistant MM cell lines, whether or not in the presence of bone marrow stem cells 63. No cytotoxicity was observed in CD38‐negative cells treated with daratumumab, confirming that induction of ADCC depends on target expression. Daratumumab induced ADCC against patient‐derived MM cells, whereby maximal killing differed per tumor sample and depended partly on the frequency of effector cells 63, 69. Importantly, daratumumab also induced effective lysis in a subset of patient‐derived MM cells with autologous effector cells, which was enhanced when combined with lenalidomide 63, 77. In bone marrow samples from MM patients, no difference in daratumumab‐mediated ADCC was observed between newly diagnosed or relapsed or refractory patients 69. The heterogeneity in responsiveness of primary MM cells toward daratumumab was associated with CD38 expression 69.

### Antibody‐dependent cellular phagocytosis

ADCP of IgG1‐opsonized cancer cells occurs via binding to FcγRs, in particular via the low‐affinity FcγRIIa and FcγRIIIa 78. Macrophages are specialized phagocytes and are an abundant component of the tumor microenvironment, including of MM 79, 80. In co‐cultures of macrophages and various MM and Burkitt's lymphoma‐derived tumor cell lines, daratumumab efficiently induced macrophage‐mediated phagocytosis, as observed in an *in vitro* flow cytometry assay 81. Interestingly, in live cell imaging studies, it was observed that individual macrophages sequentially engulfed multiple tumor cells within a relatively short time span 81. Furthermore, in *in vivo* subcutaneous and intravenous leukemic xenograft mouse models, daratumumab induced significantly stronger anti‐tumor activity compared to a matched IgG2 isotype equivalent incapable of inducing phagocytosis by mouse macrophages. This indicates that phagocytosis contributes to the mechanism of action of daratumumab *in vivo*. Importantly, daratumumab‐dependent phagocytosis was demonstrated *ex vivo* in 11 of 12 patient‐derived MM cell isolates with variable levels of CD38 expression 81, suggesting that phagocytosis is a clinically relevant mechanism of action that may contribute to the therapeutic activity of daratumumab.

### Apoptosis

Emerging evidence indicates that FcγR‐mediated cross‐linking of tumor‐bound monoclonal antibodies may induce programmed cell death (PCD) in tumor cells 82, 83, 84, 85. Indeed, in a model system in which cross‐linking of daratumumab is induced with an anti‐Fc antibody or by cells expressing FcγRI was found to kill CD38‐positive MM tumor cell lines *in vitro* via programmed cell death (PCD) (M.B. Overdijk, J.H.M. Jansen, M. Nederend, J.J. Lammerts van Bueren, R.W.J. Groen, P.W.H.I. Parren, J.H.W. Leusen, P. Boross, submitted).

Furthermore, mouse studies indicated that FcγR‐mediated cross‐linking of daratumumab also contributes to PCD induction *in vivo*. Studies in Fc*γ*R‐chain knockout (Fc*γ*R^−/−^) mice showed that expression of the inhibitory mouse FcγRIIb was sufficient to induce PCD *in vivo*, albeit at relatively low levels. Daratumumab also induced PCD of CD38‐expressing tumor cells in the NOTAM mouse model (M.B. Overdijk, J.H.M. Jansen, M. Nederend , J.J. Lammerts van Bueren, R.W.J. Groen, P.W.H.I. Parren, J.H.W. Leusen, P. Boross, submitted), which have normal surface expression of all activating murine FcγRs, but lack signaling capacity. This indicates that activating FcγRs can also mediate cross‐linking of daratumumab resulting in induction of PCD. Taken together, *in vitro* and *in vivo* data indicate that FcγR‐mediated cross‐linking of daratumumab results in PCD of CD38 expressing MM tumor cells. The underlying cellular mechanism and the contribution to the overall anti‐tumor effect *in vivo* still need to be elucidated.

### Anti‐tumor activity of daratumumab in bone marrow mononuclear cells *ex vivo*

To evaluate the cytotoxic effect of daratumumab in a more physiological system, assays were performed with bone marrow mononuclear cells from MM patients. In these assays, effector cells, tumor‐supporting stromal cells, and 2–50% malignant plasma cells were present. In this *ex vivo* setup, daratumumab induced substantial tumor cell lysis, which was significantly increased when it was combined with emerging anti‐myeloma drugs and drug combinations, including lenalidomide and bortezomib 77, 86. Various mechanisms may contribute to the cytotoxic effect of daratumumab in this setup, including induction of PCD and in particular ADCC. Of interest, addition of complement in this assay even further enhanced daratumumab‐mediated killing of myeloma cells by induction of CDC 77.

### Enzymatic modulation

**Figure 2:**
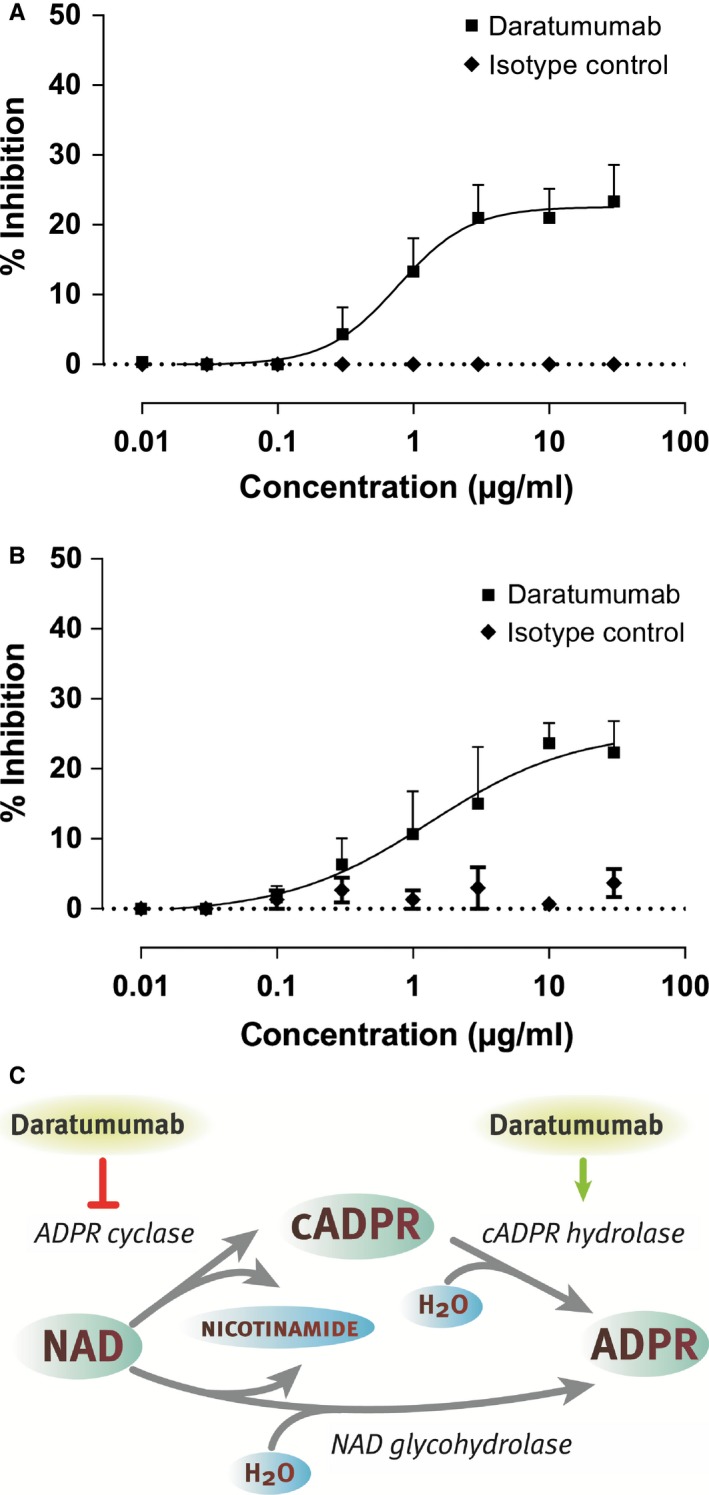
**Daratumumab modulates the enzymatic activity of** **CD** **38.** (A, B) Concentration‐dependent inhibition of CD38 cyclase activity by daratumumab tested on recombinant CD38 protein (A) or CHO cells stably transfected with human CD38 (B). Therefore, recombinant CD38 protein or CHO‐CD38 cells were incubated with daratumumab and NGD, and the presence of fluorescent cGDPR in supernatant was measured. The graphs show the % inhibition in cGDPR production compared to the untreated controls. (C) Enzymatic processing of NAD and NADP by CD38 and modulation of this activity by daratumumab.

As described above, CD38 has enzymatic activity and possesses both cyclase and hydrolase activities. In experiments using recombinant CD38 protein or CD38‐expressing cells, we observed that daratumumab inhibits the cyclase activity of CD38 *in vitro* (*Fig.* 2 *A, B*). Furthermore, daratumumab was found to stimulate the activity of CD38 to hydrolyze cADPR *in vitro* (unpublished data). Thus, daratumumab modulates the enzymatic activities of CD38, blunting cyclase activity, and enhancing hydrolase activity. Overall this will result in increased levels of NAD and ADPR and decreased cADPR concentrations (*Fig.* 2 *C*). Reduction in cADPR levels may lead to decreased Ca^2+^ mobilization and reduced signaling. Furthermore, increased NAD levels may induce cell death, although as an added complexity it should be noted that altered NAD homeostasis has multifaceted effects on cellular survival depending on cellular context 87. It has been postulated that the metabolic activity of CD38 contributes to a microenvironment that is favorable for tumor survival in closed environmental systems, such as the bone marrow niche 88. Interestingly, the CD38 enzymatic activity may also contribute to immune suppression observed in myeloma patients 88. The complex role of CD38 in calcium signaling and NAD metabolism thus complicates pinpointing the exact consequences of enzymatic modulation of CD38 by daratumumab.

### Conclusion

Thus, daratumumab is a CD38‐specific antibody that showed strong anti‐tumor activity in tumor models *in vivo* and engages multiple mechanisms of action. The antibody has a unique binding profile and effectively triggers Fc‐dependent effector functions, including CDC, ADCC, and ADCP, while Fc cross‐linking induces PCD of daratumumab‐opsonized cells. Furthermore, daratumumab modulates the enzymatic activities of CD38, blunting the cyclase activity, and enhancing the hydrolase activity. While all of these actions are involved in the anti‐tumor activity mediated by daratumumab, the exact contribution of each mechanism to the observed clinical activity needs to be elucidated.

## Preclinical rationale for daratumumab‐based combination therapies in multiple myeloma

Combinations of therapeutic agents are often superior in terms of depth of response and survival, when compared to treatment with a single agent. Combination therapies simultaneously target multiple pathologic pathways and prevent escape and resistance mechanisms of tumor cells. This also applies for combinations of pharmacological agents with therapeutic monoclonal antibodies, which often result in superior effects compared to monotherapy. Indeed, the combination of monoclonal antibodies targeting cell surface antigens on tumor cells with conventional chemotherapy or novel agents is already standard‐of‐care in several other hematologic malignancies such as non‐Hodgkin's lymphoma and CLL as well as in solid tumors including breast cancer and colon carcinoma 89.

Preclinical studies are of great importance to develop CD38‐targeting antibody‐based combination therapies with conventional drugs that have synergistic mechanisms of action and non‐overlapping toxicities. Also modulation of determinants of sensitivity of the tumor cell toward CD38‐targeting antibodies with novel therapeutic approaches may lead to more effective regimens with increased quality of response and improvement in survival.

### Immunomodulatory agents

Lenalidomide is an amino‐substituted derivative of thalidomide with direct anti‐proliferative and cytotoxic effects on the myeloma tumor cell, as well as anti‐angiogenic activity and potent immunomodulatory effects 90. Lenalidomide has remarkable activity in patients with newly diagnosed MM 91 and relapsed refractory MM 92, 93 and it has contributed to significantly improved survival of myeloma patients 94.

Because of the strong immunostimulating effects of lenalidomide including activation of NK cells (important effector cells in ADCC), we have evaluated the activity of lenalidomide combined with CD38‐targeting antibodies in diverse preclinical models. Lenalidomide significantly enhanced daratumumab‐mediated killing of MM cell lines and primary MM cells by activating the effector cells of ADCC 77. Indeed, pretreatment of effector cells with lenalidomide significantly improved their ability to kill MM cells in ADCC assays 77. Daratumumab‐mediated ADCC was also significantly upregulated when peripheral blood mononuclear cells were used from lenalidomide‐treated patients 77, whereas daratumumab‐mediated CDC, in contrast, was not affected by lenalidomide 77. Importantly, the combination of daratumumab and lenalidomide also significantly improved ADCC against MM cells derived from lenalidomide‐refractory patients 95. Finally, lenalidomide synergized with daratumumab in killing primary MM cells from a lenalidomide‐refractory patient in a humanized mouse model 95. This indicates that despite resistance of the tumor cells to lenalidomide, the patients’ immune system may still be able to respond to the immunomodulatory effects of lenalidomide. Similarly, the activity of isatuximab and MOR202 is synergistically enhanced by lenalidomide 96, 97. The activity of daratumumab or the combination of daratumumab with lenalidomide was further augmented by IPH2102, which improves NK cell activity by blocking the three main inhibitory KIR receptors (KIR2DL1/2/3) present on the cell surface of NK cells 98.

Pomalidomide is a next‐generation oral immunomodulatory agent, which has potent direct anti‐myeloma activity, inhibits stromal cell support, and appears to have stronger immune modulatory effects compared to lenalidomide 99, 100. Pomalidomide is active in relapsed/refractory myeloma patients who have received multiple lines of therapy. Importantly, pomalidomide plus low‐dose dexamethasone has also shown activity in patients with lenalidomide and bortezomib‐refractory disease with at least partial response (PR) in approximately 25–30% 101, 102, 103. *In vitro*, isatuximab‐mediated ADCC against MM cells was enhanced more potently by pomalidomide, when compared to lenalidomide 96, 104. Pomalidomide was also more effective than lenalidomide in enhancing isatuximab‐induced apoptosis 96.

### Proteasome inhibitors

Bortezomib is a potent reversible proteasome inhibitor with high anti‐MM activity. Bortezomib induces clinically significant responses with acceptable toxicity in newly diagnosed and in relapsed/refractory myeloma 105, 106. Together with the introduction of the immunomodulatory drugs, bortezomib has significantly improved the survival of patients with relapsed and refractory myeloma 94.

The combination of daratumumab and bortezomib resulted in additive effects in ADCC assays in our *in vitro* assay setup using bone marrow samples from MM patients 95. Synergistic anti‐tumor activity was also demonstrated between isatuximab and bortezomib in a myeloma mouse model 107.

### All‐trans retinoic acid

We have shown that the level of cell surface expression of CD38 on MM cells was positively associated with extent of ADCC as well as CDC 69. Interaction of all‐trans retinoic acid (ATRA) with nuclear retinoic acid receptors results in induction of CD38 expression 69, 108, 109, which significantly improved daratumumab‐mediated ADCC and CDC against MM cells 69. ATRA enhanced CDC to a larger extent than ADCC, which may be explained by concomitant downregulation of complement‐inhibitory proteins CD55 and CD59 69. Interestingly, ATRA significantly improved the anti‐MM effect of daratumumab in a humanized mouse model *in vivo* 69.

### Standard‐of‐care regimens

The three‐drug regimens MPV (melphalan–prednisone–bortezomib) 110 and RVD (lenalidomide–bortezomib–dexamethasone) 111 are widely used in the treatment of MM patients. However, patients eventually develop resistance to these three‐drug regimens. In preclinical studies, daratumumab improved the anti‐myeloma effect of both RVD and MPV 86. This indicates that addition of daratumumab to these important treatment regimens holds a significant potential to improve the clinical outcome of MM patients.

## Clinical studies in multiple myeloma

Preclinical studies by us and others provided a strong rationale of evaluating a CD38‐targeting antibody alone or in combination in MM patients with relapsed/refractory or newly diagnosed disease (*Tables* *1*, *2*, *3*).

**Table 1:** Clinical studies with daratumumab

Study	Phase	Disease	Setting	Treatment	Status*
NCT00574288 (GEN501)	1/2	MM	Relapsed/refractory	Daratumumab as single agent	Ongoing, not recruiting
NCT01985126 (SIRIUS; MMY2002)	2	MM	Relapsed/refractory	Daratumumab as single agent	Ongoing, not recruiting
NCT01615029 (GEN503)	1/2	MM	Relapsed/refractory	Daratumumab combined with lenalidomide–dexamethasone	Ongoing, not recruiting
NCT02076009 (POLLUX)	3	MM	Relapsed/refractory	Lenalidomide–dexamethasone versus lenalidomide–dexamethasone plus daratumumab	Ongoing, not recruiting
NCT02136134 (CASTOR)	3	MM	Relapsed/refractory	Bortezomib–dexamethasone versus bortezomib–dexamethasone plus daratumumab	Ongoing, not recruiting
NCT02519452	1	MM	Relapsed/refractory	Daratumumab combined with rHuPH20 in subcutaneous formulation	Not yet open
NCT01998971	1b	MM	Relapsed/refractory and newly diagnosed	Daratumumab combined with pomalidomide–dexamethasone (RR), carfilzomib–dexamethasone (RR), bortezomib–dexamethasone (ND), bortezomib–thalidomide–dexamethasone (ND), bortezomib–melphalan–prednisone (ND) or carfilzomib–lenalidomide–dexamethasone (ND)	Recruiting
NCT02252172 (MAIA)	3	MM	Newly diagnosed, non‐transplant eligible	Lenalidomide–dexamethasone versus lenalidomide–dexamethasone plus daratumumab	Recruiting
NCT02195479 (ALCYONE)	3	MM	Newly diagnosed, non‐transplant eligible	Bortezomib–melphalan–prednisone versus bortezomib–melphalan–prednisone with daratumumab	Recruiting
NCT02541383 (CASSIOPEIA)	3	MM	Newly diagnosed, transplant eligible	Randomization 1: bortezomib–thalidomide–dexamethasone induction therapy – high‐dose melphalan plus autologous stem cell rescue – bortezomib–thalidomide–dexamethasone consolidation versus bortezomib–thalidomide–dexamethasone with daratumumab induction therapy – high‐dose melphalan plus autologous stem cell rescue – bortezomib–thalidomide–dexamethasone with daratumumab consolidation Randomization 2: daratumumab as single agent in maintenance versus observation only	Recruiting
NCT02316106 (CENTAURUS)	2	Smoldering MM	Not previously treated	Daratumumab as single agent	Recruiting
NCT02413489 (CARINA)	2	CD38‐positive mantle cell lymphoma, diffuse large B‐cell lymphoma, or follicular lymphoma	Relapsed/refractory	Daratumumab as single agent	Recruiting

ND, newly diagnosed; RR, relapsed/refractory; MM, multiple myeloma.

*As of November 23, 2015.

**Table 2:** Clinical studies with isatuximab

Study	Phase	Disease	Setting	Treatment	Status*
NCT01084252	1/2	CD38‐positive hematologic malignancies including NHL, MM, AML, ALL, and CLL	Relapsed/refractory	Isatuximab as single agent	Recruiting
NCT01749969	1b	MM	Relapsed/refractory	Isatuximab in combination with lenalidomide–dexamethasone	Recruiting
NCT02283775	1b	MM	Relapsed/refractory	Isatuximab in combination with pomalidomide–dexamethasone	Recruiting
NCT02513186	1	MM	Newly diagnosed, non‐transplant eligible	Isatuximab in combination with CyBorD	Recruiting
NCT02332850	1b	MM	Relapsed/refractory	Isatuximab combined with carfilzomib	Recruiting

NHL, non‐Hodgkin's lymphoma; MM, multiple myeloma; AML, acute myeloid leukemia; ALL, acute lymphoblastic leukemia; CLL, chronic lymphocytic leukemia; CyBorD, cyclophosphamide, bortezomib, and dexamethasone. *As of November 23, 2015.

**Table 3:** Clinical study with MOR202

Study	Phase	Disease	Setting	Treatment	Status*
NCT01421186	1/2	MM	Relapsed/refractory	MOR202 with or without dexamethasone (later stage MOR202 with lenalidomide–dexamethasone and MOR202 with pomalidomide–dexamethasone)	Recruiting

*As of November 23, 2015.

### Daratumumab in relapsed/refractory MM

Based on the preclinical activity of daratumumab, a phase 1/2 study was initiated in MM patients with relapsed/refractory disease (GEN501 study) 112. In the first‐in‐human dose‐escalation part of the study, the maximum‐tolerated dose was not reached with dose levels up to 24 mg/kg. In the phase 2 part of the study, patients with a median of 4 prior lines of therapy (majority refractory to lenalidomide and bortezomib) were treated with daratumumab at a dose of 8 mg/kg or 16 mg/kg. The overall response rate (at least PR) was 36% in the 16 mg/kg cohort and 10% in the 8 mg/kg group. Remarkably, in this extensively pretreated group of patients, 2 of 42 patients treated with 16 mg/kg daratumumab achieved a first complete response. The median PFS in the 8 mg/kg and 16 mg/kg groups were 2.4 and 5.6 months, respectively. The 12‐month survival was 77% for both groups. The most frequent adverse events were infusion‐related reactions, which occurred in 71% of patients. The majority of these reactions included grade 1 and 2, and characterized by rhinitis, cough, headache, pyrexia, and dyspnea. The most infusion‐related reactions occurred during the first daratumumab infusion and only few patients (<10%) had infusion‐related reactions with more than one infusion.

The SIRIUS (MMY2002) study confirmed the results from GEN501 study that demonstrated single agent activity of daratumumab with a favorable toxicity profile 113. One hundred and six patients, with a median of 5 prior lines of therapy (95% refractory to lenalidomide and bortezomib), received daratumumab monotherapy at a dose of 16 mg/kg. At least a PR was achieved in 29% of patients with stringent complete response (CR) in 3%. The median duration of response was 7.4 months. The median progression‐free survival was 3.7 months and 1‐year overall survival was 65%. Notably, subgroup analysis showed that in the group of patients who were refractory to lenalidomide, pomalidomide, bortezomib, and carfilzomib, PR or better was achieved in 21% of these patients. Infusion‐related reactions were observed in 43% of the patients and were predominantly grade 1 and 2, and could be managed with interruption of the infusion or extra corticosteroids and anti‐histamines.

The preclinical evidence showing that lenalidomide enhances daratumumab‐mediated anti‐MM activity formed the rationale for an ongoing phase 1/2 study of daratumumab and lenalidomide plus dexamethasone (GEN503) in relapsed/refractory myeloma. Preliminary results showed high response rates (at least VGPR in 60% of patients treated with ≥4 cycles in part 2) that improved over time 114. Based on these results, a phase 3 randomized study is comparing lenalidomide–dexamethasone with or without daratumumab in the relapsed/refractory setting (POLLUX study; enrollment completed). Also the combination of daratumumab with pomalidomide and dexamethasone is evaluated in relapsed/refractory patients and preliminary results are promising with encouraging activity and little additional toxicity, other than infusion‐related reactions 115.

The combination of daratumumab with bortezomib is evaluated in the relapsed/refractory setting in the ongoing, randomized phase 3 CASTOR study, which compares bortezomib–dexamethasone with or without daratumumab in MM patients with at least one prior line of therapy (enrollment completed September 2015). A phase 1b study is also evaluating the safety of daratumumab in combination with carfilzomib and dexamethasone in relapsed/refractory patients.

### Isatuximab in relapsed/refractory MM

A first‐in‐human, phase 1 dose‐escalation study, evaluated single agent isatuximab in 34 patients with relapsed/refractory CD38‐positive malignancies 116, 117. The maximum‐tolerated dose was not reached with dosing up to 20 mg/kg. The majority of the patients in this trial had MM, and patients were heavily pretreated with a median of 6 prior regimens. In the group of MM patients treated with isatuximab at a dose of ≥10 mg/kg (*n* = 18), PR or better was observed in 33% including CR in 11%. With preinfusion prophylaxis consisting of corticosteroids, anti‐histamines, and acetaminophen, infusion‐related reactions occurred in 39% of the patients who were treated with isatuximab at a dose of ≥3 mg/kg. All infusion‐related reactions occurred during the first infusion and were predominantly grade 1 or 2. Symptoms of infusion‐related reactions included nausea, pyrexia, vomiting, dyspnea, cough, and nasal congestion.

Based on the observed single agent activity of isatuximab and preclinical evidence of synergy between isatuximab and lenalidomide, the combination of isatuximab and lenalidomide–dexamethasone is currently evaluated in a phase 1 clinical study with relapsed/refractory MM patients 116. Preliminary results show that the combination is effective and has a favorable toxicity profile 116. Interestingly, response was also achieved in lenalidomide‐refractory patients (PR or better: 48%), which suggests that the immune system of these patients could still respond to the immunomodulatory effects of lenalidomide 116. Infusion‐related reactions were observed in 39% of the patients, predominantly during cycle 1. Other ongoing studies in the relapsed/refractory setting include isatuximab with pomalidomide–dexamethasone or carfilzomib (*Table* 2).

### MOR202 in relapsed/refractory MM

An ongoing phase 1/2 study in relapsed/refractory myeloma (median of 4 prior therapies) showed that MOR202 was well tolerated and the maximum‐tolerated dose has not yet been reached 118. Infusion‐related reactions occurred in 31% of patients receiving MOR202 without dexamethasone, mainly during the first infusion 118, 119. There were no infusion‐related reactions when MOR202 was combined with dexamethasone 118. In early cohorts, long‐lasting tumor control was observed 118. At a later stage, the combination of MOR202 with lenalidomide–dexamethasone and pomalidomide–dexamethasone will also be evaluated in the same study 118, 119.

### CD38‐targeting antibodies in newly diagnosed MM

Based on the efficacy and favorable toxicity profile of the CD38‐targeting antibodies in patients with relapsed/refractory MM, these agents are currently also evaluated in patients with newly diagnosed MM.

The MAIA study is a randomized phase 3 study, which will compare lenalidomide plus dexamethasone with or without daratumumab in the setting of newly diagnosed disease in MM patients not eligible for transplantation. In addition, the randomized phase 3 ALCYONE study will compare bortezomib–melphalan–prednisone (VMP) with VMP plus daratumumab in previously untreated MM patients who are ineligible for autologous stem cell transplantation 120. Furthermore, the HOVON and IFM study groups have already started enrolling newly diagnosed MM patients with age of ≤65 years in the Cassiopeia phase 3 randomized trial. In this study patients will be treated with VTD (bortezomib–thalidomide–dexamethasone) with or without daratumumab as induction therapy, followed by high‐dose melphalan with autologous stem cell rescue, and then 2 cycles of VTD with or without daratumumab as consolidation. A second randomization will evaluate the value of daratumumab in maintenance versus observation only. In addition, daratumumab is evaluated with KRD (carfilzomib–lenalidomide–dexamethasone) in newly diagnosed MM patients.

Isatuximab will also be evaluated in the setting of newly diagnosed disease in combination with cyclophosphamide, bortezomib, and dexamethasone (CyVD).

### Smoldering MM

Smoldering MM (SMM) is a precursor state of MM, characterized by M‐protein ≥30 g/l or bone marrow plasma cell percentage of 10–60% without end‐organ damage or presence of biomarkers of malignancy 121, 122. Patients with SMM are not treated until symptomatic disease develops. SMM is a heterogeneous clinical entity with a subset of patients at high‐risk of progression (rate of progression: approximately 70% at 5 years) 123, 124. Importantly, a recent study in high‐risk SMM showed that lenalidomide–dexamethasone delayed progression to active disease and improved survival, when compared to observation only 125. Since the presentation of these promising results, several other studies in SMM have been initiated including trials with monoclonal antibodies such as siltuximab (anti‐IL6), IPH2102 (anti‐KIR2D) 126, and elotuzumab (anti‐SLAMF7), as well as daratumumab as a single agent in three different dosing schedules (CENTAURUS study).

### Host‐ and tumor‐related factors that predict response to CD38‐targeting antibodies

We have demonstrated that CD38 expression levels are associated with the efficacy of daratumumab to induce ADCC and CDC in *ex vivo* experiments, and also with response to daratumumab as single agent in the GEN501 and SIRIUS studies 69, 127. Furthermore, we showed that during treatment there was a significant decrease in CD38 expression on residual bone marrow‐localized MM cells as well as circulating tumor cells 127. Interestingly, in these two daratumumab monotherapy studies, progressive disease was associated with upregulation of the complement‐inhibitory proteins CD55 and CD59 127. Expression of these complement inhibitors was not different between responders and non‐responders before start of treatment 127. The role of CD38 and complement inhibitors as determinants of depth of response and development of resistance has to date not been described for MOR202 and isatuximab.

Variation in genes involved in the immune response may also be associated with response to CD38‐targeted therapies. Indeed, KIR and HLA genotypes predictive of high‐affinity NK cell receptor–ligand interactions were associated with improved clinical outcome in MM patients treated with isatuximab combined with lenalidomide and dexamethasone 128.

## Other applications

### Other CD38‐positive hematologic malignancies

CD38 is not only expressed on MM cells but also in other hematologic malignancies derived from both lymphoid and myeloid lineages including non‐Hodgkin's lymphoma 129, acute myeloid leukemia 55, acute lymphoblastic leukemia 55, as well as in CLL. However, the prevalence and expression levels of CD38 in these tumors are much lower compared to MM. For instance, CD38 expression is found in 27–46% of CLL cases and has been associated with aggressiveness of disease and worse patient outcome 51, 130, 131. Preclinical evidence shows that the CD38‐targeting antibodies have activity against these CD38‐positive malignancies.

The CD38 antibodies daratumumab and MOR202 induced potent killing of CD38‐expressing lymphoma cells primarily via ADCC and ADCP 63, 132, 133, 134, 135. Similarly, isatuximab kills lymphoma cells via ADCC and ADCP, and also has strong proapoptotic activity in lymphoma in the absence of cross‐linking agents 64. Daratumumab also shows preclinical anti‐tumor activity against CD38‐expressing CLL tumor cells and inhibits adhesion and migration of CLL tumor cells in the tumor microenvironment 133, 134, indicating that CD38‐targeting antibodies may also be of value in the treatment of CD38‐positive CLL. Due to relatively high expression of the complement inhibitors and lower CD38 expression levels compared to MM cells, daratumumab failed to effectively induce CDC in most patient‐derived NHL and CLL cells 133, 134. Nevertheless, daratumumab and isatuximab showed a marked anti‐tumor activity in mouse xenograft models of NHL *in vivo* 63, 64, 134. Based on these results, daratumumab is being evaluated in a phase 2 clinical trial in patients with CD38‐positive mantle cell lymphoma, diffuse large B‐cell lymphoma, or follicular lymphoma (*Table* 1). As only a subset of NHL patients shows CD38 expression, patients included in this study are selected based on CD38 expression. A small number of patients with non‐Hodgkin's lymphoma were also included in the phase 1/2 study with isatuximab monotherapy. However, so far outcome of these patients has not been separately reported 117.

In preclinical models, daratumumab effectively inhibited the growth of ALL tumors that express high levels of CD38 136. Similarly, isatuximab was highly efficacious in a B‐ALL xenograft model 64, while CD38‐targeting antibodies have also shown activity against T‐ALL cells 64. In acute myeloid leukemia (AML) xenograft models, daratumumab significantly reduced leukemia burden in peripheral blood and spleen, but had no effect in the bone marrow, which suggests that the bone marrow microenvironment can impair the anti‐leukemic activity of daratumumab 137. In contrast, studies in MM models suggested that daratumumab can also effectively mediate anti‐tumor activity in the bone marrow environment 63, 77, 86, 138. The precise role of the bone marrow microenvironment in daratumumab susceptibility remains to be elucidated. Finally, ongoing studies are testing the combination of daratumumab with chemotherapeutic drugs such as Ara‐C and doxorubicin in AML mouse models 137.

### Immunomodulation

A recent study showed that a CD38 mAb released T‐cell suppression by CD38‐expressing myeloid‐derived suppressor cells (MDSCs) resulting in a decreased tumor growth rate in an *in vivo* model of esophageal cancer 39. Increased numbers of CD38‐positive MDSCs were also observed in the blood of patients with advanced head and neck and non‐small cell lung cancer 39. Myeloid‐derived suppressor cells and a subset of regulatory T‐cells (CD38+ Treg) have susceptibility to daratumumab‐mediated cell lysis (Darzalex, US prescribing information). These results are encouraging and further studies toward the potential immunomodulatory activity of CD38‐targeting antibodies are warranted, including for hematological and solid tumors that are CD38 negative.

### Autoimmune disorders

Autoantibody production by plasma cells plays an important role in the pathogenesis of several autoimmune diseases such as systemic lupus erythematosus (SLE), vasculitis, autoimmune cytopenias, and rheumatoid arthritis. B‐cell depleting therapy with anti‐CD20 or anti‐CD22 monoclonal antibodies is frequently used in autoimmunity to reduce autoantibody production, antigen presentation, cytokine production, and activation of T cells 139, 140, 141. However, long‐lived autoreactive plasma cells largely resist B‐cell‐targeted therapy, and these non‐eradicated plasma cells continue to produce autoantibodies 141, 142. Recently, it was shown that CD19‐negative bone marrow plasma cells, which express CD38, are enriched in chronically inflamed tissue and secrete autoantibodies 143.

While CD38‐targeting antibodies were initially developed to kill malignant plasma cells, these monoclonal antibodies may also abrogate the production of autoantibodies in autoimmune disorders and thereby reduce autoantibody‐dependent effector mechanisms. Therefore, treatment of patients with SLE and other autoimmune disorders with anti‐plasma cell antibodies alone or in combination with standard‐of‐care therapies, may be a new therapeutic approach. However, at this moment there is no preclinical or clinical data available to estimate the impact of plasma cell‐directed therapy with CD38‐targeting antibodies on humoral autoimmunity.

Similarly, CD38 antibodies may mediate depletion of allergen‐specific IgG, A, or E secreting plasma cells that maintain inflammatory processes. By employing this anti‐inflammatory action, anti‐CD38 therapy may be beneficial in patients with severe allergic conditions. Furthermore, in transplantation medicine reduction in alloantibody levels with an anti‐CD38 antibody during acute rejection may diminish transplant loss 142.

## Resolving interference of daratumumab in clinical laboratory assays

Interference of drugs in clinical laboratory tests is a well‐known problem 144. In particular therapeutic mAbs, which are increasingly used in the clinic, are prone to interfere in laboratory tests, as the selective antibody–antigen interactions of therapeutic mAbs are sometimes similar to the interactions employed by clinical laboratory tests. CD38 antibodies appear to interfere with clinical laboratory tests, the indirect antiglobulin test (IAT), and serum protein and immunofixation electrophoresis assays, causing unexpected false‐positive results. Various methods have been developed to neutralize the interference of daratumumab in these assays.

### Indirect antiglobulin test

In clinical trials with daratumumab, some patients required a blood transfusion (unrelated to daratumumab treatment). All patients receiving daratumumab showed positive IATs, which is used for the detection of irregular blood group antibodies against donor red blood cells 145, 146. These false‐positive test results were caused by a fraction of red blood cells, which appeared to express a low level of CD38.

**Figure 3:**
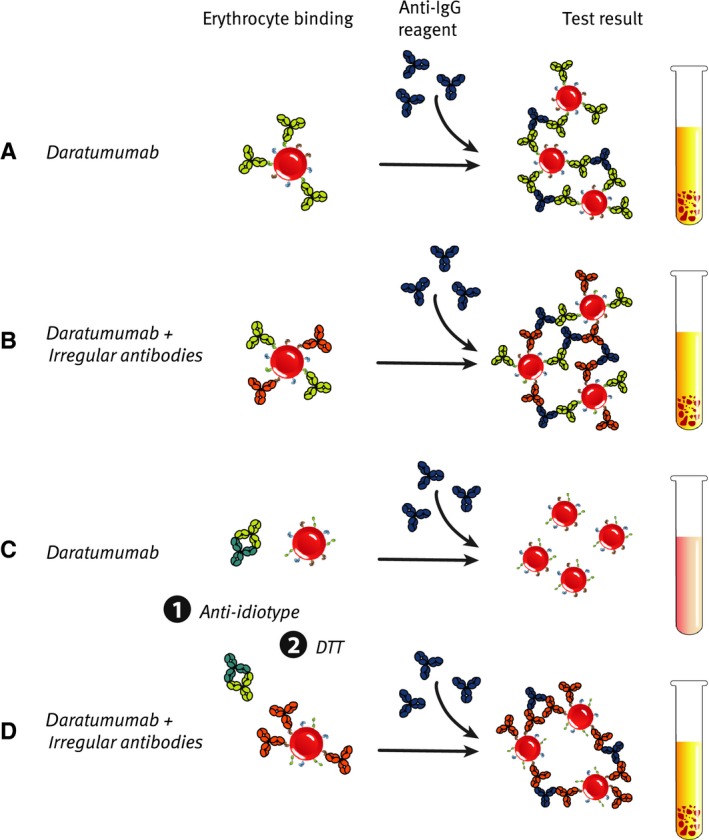
**Resolving interference of daratumumab in the indirect antiglobulin test (** **IAT** **).** (A, B) Daratumumab in the patient's serum binds to the test RBCs. After adding the anti‐IgG reagent, RBC agglutination is observed, thereby generating a false‐positive result (A) or masking the presence of irregular antibodies (B). (C, D) Daratumumab‐specific anti‐idiotype antibodies are added to the patient's serum and bind to daratumumab (1). Alternatively, test RBCs are treated with DTT, resulting in denaturation of CD38 and loss of daratumumab binding (2). If the patient has no irregular antibodies, binding of daratumumab to RBCs is blocked generating a negative IAT test result. However, if the serum contains irregular antibodies, binding of these antibodies to RBC will result in a true positive IAT test result.

A number of methods were established to neutralize the interference of daratumumab in the blood typing test so that proper identification of optimal donor blood is reestablished (*Fig.* 3). Addition of a daratumumab‐specific anti‐idiotype antibody or soluble CD38 (sCD38) antigen to the IAT plasma sample neutralized the interference of daratumumab 145, 146. Alternatively, treatment of reagent red blood cells with the reducing agent DTT also abrogated the interference of daratumumab in the IAT 145. In the latter method, reduction in the disulfide bonds that are present in the extracellular domain of CD38 16 results in denaturation of the CD38 protein and abolishes binding of CD38 mAbs 147. A drawback of this method is that DTT denatures also a limited number of other blood group antigens, including Kell antigens 145, but this can be managed by providing K‐negative units to these patients 145. Advantages of the CD38 denaturation method are the widespread availability of DTT in blood banks worldwide and the low costs.

### Serum protein electrophoresis (SPEP) and immunofixation electrophoresis (IFE)

A known problem is the interference of therapeutic mAbs in assays that monitor clonal myeloma protein (M‐protein) as a measure for disease burden in myeloma patients 148. Daratumumab is also detected on serum protein electrophoresis (SPEP) and immunofixation (IFE) gels, and may interfere with IMWG response criteria requiring negative SPEP/IFE results to determine CR. A reflex assay was developed that uses a mouse anti‐daratumumab antibody to shift the IFE migration of daratumumab away from the range of endogenous M‐protein to be able to distinguish daratumumab from M‐protein 149. The assay was validated using daratumumab‐treated patient samples, in which M‐protein depletion could be detected without interference with the therapeutic antibody. This allowed detection of a CR under these conditions that was confirmed with additional clinical tests including bone marrow analysis 149.

Given that monoclonal antibody therapies are increasingly being used to treat a wide range of diseases, this reflex assay offers a potential solution to SPEP and serum IFE interference that occurs with a number of other therapeutic antibodies.

## Conclusions and future perspectives

The multifunctional cell surface protein CD38 possesses receptor as well as enzyme functions. CD38 is expressed in a subset of hematological tumors, and shows particularly broad and high expression levels in MM. Together, this pointed to CD38 as an attractive therapeutic target and triggered the development of various therapeutic CD38 antibodies, including daratumumab, isatuximab, and MOR202. These CD38‐targeting antibodies have a favorable toxicity profile in patients and early clinical data show a marked activity in MM.

Recent advances with novel classes of drugs, including proteasome inhibitors and immunomodulatory agents, have significantly improved the prospects of MM patients. However, the vast majority of patients will eventually develop disease that is refractory to all available agents, which confers a very poor prognosis 56. Novel approaches, including immunotherapy with mAbs, are being explored to force a breakthrough and to significantly improve the outcome for this population of patients. In addition to CD38 mAbs, other therapeutic antibodies are in clinical development in MM, including antibodies directed against SLAMF7/CS1 (elotuzumab) and B cell‐activating factor (tabalumab) 150. Except for CD38 antibodies, none of these mAbs have shown anti‐myeloma activity as single agents, but enhancement of the anti‐tumor efficacy was noted in combination with other agents 62. Also combinations of immunomodulatory agents and proteasome inhibitors with CD38 mAbs show much promise in preclinical studies and early clinical data. Many clinical studies are currently evaluating daratumumab and other CD38 mAbs in combination with various different anti‐myeloma regimens (*Tables* *1*, *2*, *3*).

An improved understanding of mechanisms that contribute to innate or acquired resistance to CD38‐targeted antibodies may result in the rational design of new antibody‐based combinations with higher anti‐MM activity. Variables that have an impact on clinical outcome include host‐related factors such as frequency and activity of effector cells which may be affected by various anti‐MM therapies. Furthermore, tumor‐related factors such as target antigen expression levels, expression of complement‐inhibitory proteins, expression of inhibitory receptors such as PD‐L1, which inhibits PD‐1‐positive NK and T cells, or CD47, which impairs phagocytosis, may impact clinical outcome 96, 151. Also genetic variations in certain genes including FcγR polymorphisms 152 and KIR 128 may influence response to monoclonal antibody‐based therapy. These mechanisms are currently analyzed in detail in the studies with daratumumab and other CD38‐targeting antibodies.

In addition to MM, CD38 expression is observed in various other hematological malignancies, such as NHL and CLL, suggesting that CD38 mAbs may be active in these indications as well. However, unlike myelomas only a subset of lymphomas expresses CD38 urging the need for selection of CD38‐positive tumors that are eligible for treatment with CD38 mAbs.

Appreciating CD38 as an attractive therapeutic target in hematological malignancies, novel therapeutic approaches are being explored that target CD38, including CD38‐specific CAR T cells 153 and CD3xCD38 bispecific antibodies 67. These strategies show promise in preclinical models, but their activity in the clinic remains to be tested.

In addition, following on the success of both rituximab and trastuzumab with regard to development of subcutaneous delivery of monoclonal antibodies in oncology, subcutaneous delivery of daratumumab in combination with recombinant human hyaluronidase is currently ongoing. A subcutaneous product will offer significant advantages over IV delivery in terms of its convenience for delivery and potential side effect profile. A phase 1 study is currently ongoing in the relapsed/refractory setting of myeloma.

In conclusion, CD38 is an emerging therapeutic target for the treatment of hematological malignancies, in particular MM. The CD38 mAb daratumumab has shown encouraging activity as single agent in heavily pretreated MM patients. Daratumumab has differentiating mechanisms of action and a limited toxicity profile, which allows favorable combination therapies with existing as well as emerging therapies. It is expected that the introduction of CD38 mAbs as a novel treatment option will significantly contribute to improved outcomes of myeloma patients. Furthermore, daratumumab has therapeutic potential in indications beyond MM, in particular in other CD38‐positive hematological malignancies. Recent, exciting data indicate that CD38‐targeted inhibition of myeloid‐derived suppressor cells could mediate anti‐tumor activity through the release of T‐cell suppression. In addition to hematological tumors, this immune stimulatory potential suggests a role for CD38 antibodies in the treatment of solid tumors, however, further studies addressing this are warranted. Finally, the expression of CD38 on plasma cells may provide opportunities for the treatment of antibody‐mediated autoimmune diseases using CD38‐targeted therapies.
